# Occurrence of the Omicron variant of SARS-CoV-2 in northern Viet Nam in early 2022

**DOI:** 10.5365/wpsar.2022.13.3.955

**Published:** 2022-09-19

**Authors:** Trang thi Hong Ung, Phuong Vu Mai Hoang, Son Vu Nguyen, Hang Le Khanh Nguyen, Phuong thi Kim Nguyen, Dan Tan Phan, Thanh Thi Le, Anh Phuong Nguyen, Thach Co Nguyen,, Futoshi Hasebe, Mai thi Quynh Le

**Affiliations:** a National Influenza Center, National Institute of Hygiene and Epidemiology, Hanoi, Viet Nam.; b Department of Infection Control, 108 Military Central Hospital, Hanoi, Viet Nam.; c Division of Preventive Medicine, Viet Nam Military Medical Department, Hanoi, Viet Nam.; d Vietnam Research Station, National Institute of Hygiene and Epidemiology, Hanoi, Viet Nam.; e Institute of Tropical Medicine, Nagasaki University, Nagasaki, Japan.

## Abstract

The Omicron variant caused a surge of infections with severe acute respiratory syndrome coronavirus 2 (SARS-CoV-2) in Viet Nam in early 2022, signalling community transmission. We report on active whole-genome sequencing surveillance of positive SARS-CoV-2 samples collected at that time in northern Viet Nam from international arrivals and community clusters. We used an amplicon protocol developed with 14 polymerase chain reaction products and the Illumina iSeq 100 platform. Overall, 213 nasopharyngeal or throat swabs were analysed, of which 172 samples were identified with the Omicron variant. Of these, 80 samples were collected from community cases in February 2022, among which 59 samples were sublineage BA.2 and one sample was the recombinant XE variant. Our results indicated that Omicron had replaced Delta as the dominant variant in a very short period of time and that continuously conducting active whole-genome sequencing surveillance is necessary in monitoring the evolution and genomic diversity of SARS-CoV-2 in Viet Nam.

During the coronavirus disease (COVID-19) pandemic, Viet Nam changed its policy from “zero COVID” to “safe and flexible adaptation and effective control of the COVID-19 pandemic” (Resolution No. 128/NQ-CP, dated 11 October 2021). This was in an attempt to achieve a new normal by the end of September 2021, when the surge of the Delta (B.1.617.2) variant had decreased and was under control in southern Viet Nam. However, a surge of the Omicron (B.1.1.529) variant in early 2022 caused the highest peak of severe acute respiratory syndrome coronavirus 2 (SARS-CoV-2) infections in the country compared to previous waves.

The Omicron variant of SARS-CoV-2 was first identified in South Africa in mid-November 2021. (*1*) The first case of the Omicron variant in Viet Nam was confirmed in a person who returned to Hanoi from the United Kingdom of Great Britain and Northern Ireland on 27 December 2021. (*2*) The first community cluster of the Omicron variant (three cases) was documented in Ho Chi Minh City on 18 January 2022. Since then, a large number of cases have been detected among people arriving from overseas. As of 2 January 2022, approximately 79.2% of the population of Viet Nam had received one dose of COVID-19 vaccine and 70.1% had received two doses. However, the Omicron variant’s greater transmissibility and ability to evade immunity meant the surge of SARS-CoV-2 infections and the ensuing community transmission was expected. (*3*, *4*)

Since early 2020, the National Institute of Hygiene and Epidemiology (NIHE) has conducted active virological surveillance using whole-genome sequencing on samples positive for SARS-CoV-2. Until 15 March 2022, these samples were collected from international arrivals at quarantine centres and new community cluster infections to monitor the genomic epidemiology of SARS-CoV-2 virus circulation in northern Viet Nam. Here we report the results of complete genome sequences of SARS-CoV-2 from samples collected from 1 January to 28 February 2022 in northern Viet Nam.

## Methods

**Fig. 1:**
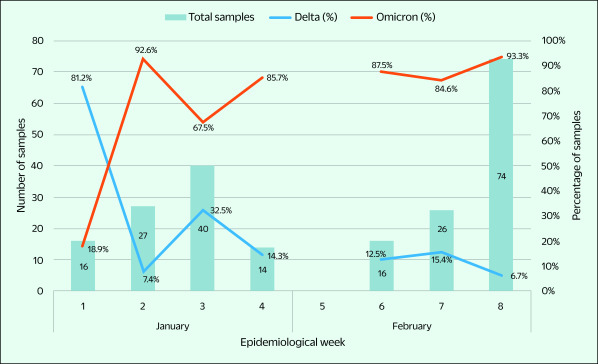
Distribution of samples sent for whole-genome sequencing by SARS-CoV-2 variant in northern Viet Nam, January and February 2022

We sequenced 213 nasopharyngeal or throat swab samples that were sent to NIHE from 22 of the 28 Provincial Centers for Disease Control and Prevention in northern Viet Nam. All samples were positive for SARS-CoV-2 with a Ct value of < 30 by real-time reverse transcription polymerase chain reaction (RT–PCR). Of the 213 specimens, 80 were collected from community outbreaks during February 2022, and the remaining 133 were from international arrivals at quarantine centres during January and February 2022 (**Fig. 1**, **Table 1**).

[**insert** Figure 1]

**Table 1:** Circulation of SARS-CoV-2 variants in samples sent for whole-genome sequencing in northern  Viet Nam, January and February 2022

Variant	January (week)					February (week)				
	1	2	3	4	Total	5^a^	6	7	8	Total
**Imported cases**										
Delta	13	2	13	2	30	Tet holiday	1	0	0	1
Omicron BA.1	3	9	15	2	29		3	0	1	4
Omicron BA.1.1	0	14	10	3	27		5	0	0	5
Omicron BA.2	0	2	2	7	11		6	5	15	26
**Community cases**										
Delta	0	0	0	0	0	Tet holiday	1	4	5	10
Omicron BA.1	0	0	0	0	0		0	1	3	4
Omicron BA.1.1	0	0	0	0	0		0	3	3	6
Omicron BA.2	0	0	0	0	0		0	13	46	59
Omicron XE	0	0	0	0	0		0	0	1	1

^a^ Samples were not collected during week 5 due to the Tet holiday.

We used the amplicon protocol developed with 14 PCR products – primers designed for sequential amplified fragments with a size band of about 2.5 kb based on the SARS-CoV-2 reference genome (ID: MN 908947-Wuhan-Hu-1). First, RNA was converted into cDNA using SuperScript^TM^ IV VILO^TM^ (Thermo Fisher Scientific, Waltham, MA, United States of America), then 14 amplicons were amplified from the cDNA using Platinum^TM^ SuperFi II Green PCR Master Mix (Thermo Fisher Scientific). Following amplification, PCR products were checked by electrophoresis using a 1% agarose gel. PCR fragments were pooled and purified by Applied Biosystems ExoSAP-IT^TM^ (Thermo Fisher Scientific). Library preparation was performed following protocol using the Nextera XT Library Preparation Kit (Illumina, San Diego, CA, United States of America) and sequencing was performed on the Illumina iSeq 100 System (Illumina). Data analysis was performed using CLC Genomics Workbench 11.0 for consensus assembly and variant detection. The sublineages were assigned using Nextclade V1.14.0 and Pangolin (lineage version 2022–02–28). (*5*, *6*) These sequences were uploaded to GISAID (IDs: EPI_ISL_11775985 to EPI_ISL_11776195).

## Results

### Sample source

Among the 213 samples in this study, 97 were collected in January 2022 (45.5%), all of which were from foreigners or returning Vietnamese citizens who were staying in quarantine centres in northern Viet Nam in accordance with immigration requirements (**Fig. 1**).

After the Vietnamese Lunar New Year (Tet holiday from 31 January to 4 February 2022), increases in SARS-CoV-2 infections in many cities and provinces in Viet Nam were reported, and samples collected from community outbreaks were sent to NIHE. We analysed a total of 116 samples in February, 80 of which came from community outbreaks in 18 cities and provinces in northern Viet Nam. Most of these (58/80; 72.5%) were collected during week 8 of 2022 (20 February). The other 36 samples were collected from quarantine centres (**Table 1**).

Two variants of concern were detected in this study. The Delta variant was at its most dominant (81.3%; 13/16) in week 1 of 2022, then decreased to 6.8% (5/74) by the last week of February. Conversely, the Omicron variant was detected in week 1 in 18.8% (3/16) of samples, then increased to 85.7% (12/14) by the last week of January (**Fig. 1**). Among the samples analysed in February 2022, the Omicron variant comprised 90.5% (105/116) overall and reached its highest rate of 93.2% (69/74) in week 8 (**Fig. 1**).

### Sublineages of SARS-CoV-2 variants detected in northern Viet Nam in early 2022

Of the 213 positive samples, 41 (19.2%) were the Delta variant. Most of these (75.6%; 31/41) were imported: 30 in January and one in early February. The other 10 Delta samples were detected in the community throughout February. The remaining 172 positive samples were the Omicron variant. We detected four Omicron sublineages: 21.5% BA.1 (37/172), 22.1% BA.1.1 (38/172), 55.8% BA.2 (96/172) and one sample (0.58%) was determined to be recombinant Omicron XE (**Table 1**). The XE variant was first detected in week 8 (on 26 February) from a community outbreak in the capital of Hanoi (GISAID ID: EPI_ISL_11776032).

### Time course of the Omicron variant in community outbreaks

Our results show that the Omicron variant was first detected in the community in week 7 of 2022 and accounted for the majority of cases in weeks 7 and 8. BA.2 was the predominant sublineage comprising most of the new COVID-19 cases in northern Viet Nam after the Tet holiday. (*2*) Co-circulating with BA.2 were the BA.1 and BA.1.1 sublineages, both of which outnumbered BA.2 among cases in January but accounted for only a small number of cases in February (**Table 1**). From week 7 to week 8, the number of BA.2 cases in the community jumped from 13 to 46, far outpacing the growth rate of other sublineages (**Table 1**).

## Discussion

As of writing, the Omicron variant is the predominant variant circulating globally. (*6*-*8*) Our findings revealed the rapid replacement of Delta by the Omicron variant in Viet Nam in January 2022, during which all positive samples were collected from people arriving in Viet Nam from abroad. Our results showed the proportion of the Omicron variant increased over time and accounted for 69.9% of infections among international arrivals in January 2022. These results corresponded to the epidemiological situation in other parts of Asia such as Hong Kong Special Administrative Region (China), Japan, the Republic of Korea and Singapore at that time. (*7*-*10*)

Among the Omicron sublineages, BA.2 was the main cause of community outbreaks. This sublineage accounted for 73.8% of analysed samples that underwent genetic sequencing and increased quickly among samples from the community (0 in week 6, 13 in week 7 and 46 in week 8), which shows the Omicron BA.2 sublineage to be a more contagious strain.

This report is the first to identify the Omicron XE variant in the community in Viet Nam. Because all imported cases were sequenced and the XE variant was not found among international arrivals, this case had no known link to the XE variant circulating in the United Kingdom at the time and may have developed independently. (*11*)

Our study has some limitations. It was conducted during the first 8 weeks of 2022 and was paused during week 5 due to the Tet holiday, so the data are not consecutive. The results were only collected in northern Viet Nam and were not representative of the entire country at the time. Additionally, this study focused on virological data, thus epidemiological data were not included. As specimens sent for whole-genome sequencing are from international arrivals and community clusters, not all notified cases are included in virological surveillance. For example, during the week of 14–20 February 2022, there were 276 633 new COVID-19 cases reported in Viet Nam, suggesting that those included in this study are a small fraction of total cases. (*12*)

Our results provide additional information about the spread of the Omicron BA.2 sublineage in Viet Nam, which may help plan for and manage infections in the near future. Active surveillance of SARS-CoV-2 variants based on whole-genome sequencing is one source of information along with other sources of surveillance that contribute to risk assessment for adjusting public health measures and assessing vaccine effectiveness.
